# Altered Structural and Functional MRI Connectivity in Type 2 Diabetes Mellitus Related Cognitive Impairment: A Review

**DOI:** 10.3389/fnhum.2021.755017

**Published:** 2022-01-06

**Authors:** Hao Lei, Rong Hu, Guanghua Luo, Tingqian Yang, Hui Shen, Hao Deng, Chunyu Chen, Heng Zhao, Jincai Liu

**Affiliations:** Department of Radiology, The First Affiliated Hospital, Hengyang Medical School, University of South China, Hengyang, China

**Keywords:** type 2 diabetes mellitus, cognitive impairment, MRI, brain connectivity, network

## Abstract

Type 2 diabetes mellitus (T2DM) is associated with cognitive impairment in many domains. There are several pieces of evidence that changes in neuronal neuropathies and metabolism have been observed in T2DM. Structural and functional MRI shows that abnormal connections and synchronization occur in T2DM brain circuits and related networks. Neuroplasticity and energy metabolism appear to be principal effector systems, which may be related to amyloid beta (Aβ) deposition, although there is no unified explanation that includes the complex etiology of T2DM with cognitive impairment. Herein, we assume that cognitive impairment in diabetes may lead to abnormalities in neuroplasticity and energy metabolism in the brain, and those reflected to MRI structural connectivity and functional connectivity, respectively.

## Introduction

Diabetes mellitus (DM) is a persistent hyperglycemia caused by insulin deficiency, insulin resistance, or both. Type 1 diabetes mellitus (T1DM) is an autoimmune disease characterized by absolute or near complete loss of insulin secretion and type 2 diabetes mellitus (T2DM) is characterized by decreased insulin sensitivity and relative insulin deficiency. T2DM is the most predominant subtype of diabetes, accounting for approximately 90% of cases (Chen et al., 2011; Hu and Jia, 2018; Zheng et al., 2018). DM and its complications can lead to cognitive impairment (Biessels et al., 2006; Cheng et al., 2012; McCrimmon et al., 2012; Gao et al., 2016; Xue et al., 2019; Lyu et al., 2020). The data show a stronger association between cognitive impairment in T2DM than T1DM (Cheng et al., 2012; Gao et al., 2016; Lyu et al., 2020). The characteristics of cognitive impairment in T2DM are the most obvious obstacle to learning and memory (Awad et al., 2004; Messier, 2005; van den Berg et al., 2010; McCrimmon et al., 2012). Over time, the decline in cognitive function can lead to faster aging of the central nervous system or increased risk of neurodegenerative diseases, such as Alzheimer's disease (AD) (Cheng et al., 2012; Verdile et al., 2015; Cheong et al., 2020; You et al., 2021). Therefore, T2DM would seriously affect the quality of life in T2DM patients, while aggravating the social burden to some extent. Here, we focus on T2DM-related cognitive impairment.

**Figure 1:**
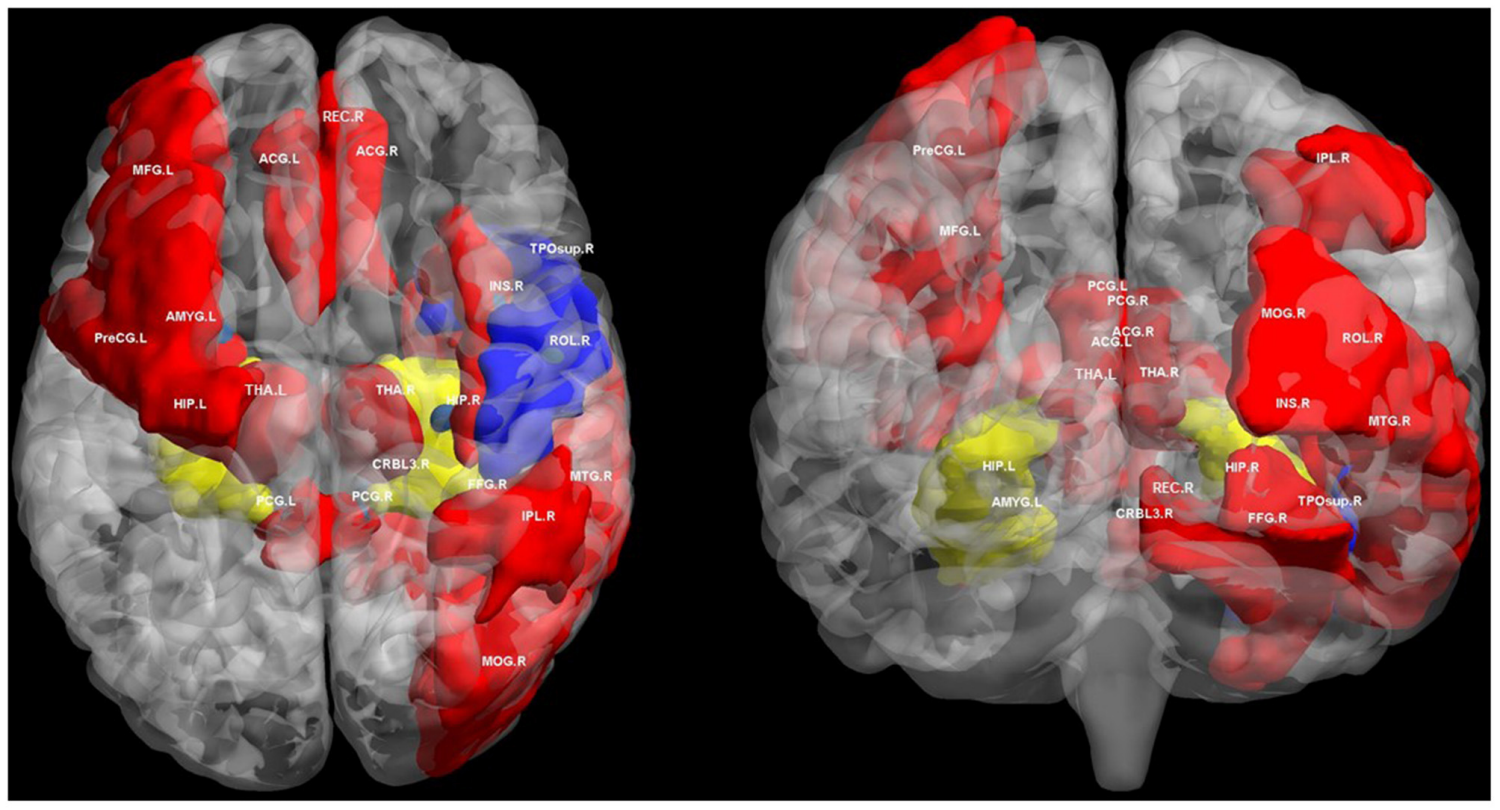
Regions of altered structural and functional connectivity in T2DM. We summarize previously reported brain regions of T2DM related cognitive impairment based on structural connectivity and functional connectivity analysis. We extracted these brain regions from automated anatomical labeling (AAL) and displayed them in BrainNet Viewer (http://www.nitrc.org/projects/bnv/). Blue regions only indicate brain regions with disrupted structural connectivity in T2DM related cognitive impairment. Red regions only indicates brain regions with disrupted functional connectivity in T2DM related cognitive impairment. The yellow regions indicate the brain area where both structural and functional connectivity was interrupted in T2DM related cognitive impairment, mainly the hippocampus. PreCG, precental gyrus; MFG, middle frontal gyrus; ROL, rolandic operculum; REC, gyrus rectus; INS, insula; ACG, anterior cingulate and paracingulate gyri; PCG, posterior cingulate gyrus; HIP, hippocampus; AMYG, amygdala; MOG, middle occipital gyrus; FFG, fusiform gyrus; IPL, inferior parietal, but supramarginal and angular gyri; THA, thalamus; TPOsup, temporal pole: superior temporal gyrus; MTG, middle temporal gyrus; CRBL3, cerebellum superior; R, right; L, left.

In neuroimaging studies, extensive changes in brain connectivity have been observed in T2DM (Reijmer et al., 2013b; Zhang et al., 2016, 2019; Chen et al., 2017; Qin et al., 2019). Previous studies based on MRI brain connectivity suggest that changes in brain structural and functional connectivity in T2DM are associated with a wide range of cognitive impairments, and maybe the overall impairment of brain connectivity (Supplementary Table 1; Figure 1). The hippocampus is one of the most important brain regions involved in T2DM related cognitive impairment, and both structural and functional connectivity disruptions are observed in it (Zhang et al., 2015, 2019; van Bussel et al., 2016a). Sun et al. suggested that reduced hippocampal functional connectivity may be closely associated with disruption of white matter integrity (Sun et al., 2018). Connections between the hippocampus and many brain regions plays critical roles in advanced cognition, including the medial temporal lobe, the medial prefrontal cortex, anterior and posterior cingulate cortex (Greicius et al., 2009; Zhou et al., 2010; Anticevic et al., 2012; Tuligenga et al., 2014; Zeidman and Maguire, 2016). Hippocampus is one of the brain structures closely related to Alzheimer's disease and has also been proved to be a vulnerable region affected by T2DM (Gold et al., 2007). Hippocampus atrophy is associated with cognitive impairment in AD (Apostolova et al., 2010). Evidence of hippocampal neuron loss was also found in diabetic rats (Xue et al., 2009). However, abnormal neuronal connectivity in T2DM may precede clinical manifestations of brain atrophy and cognitive impairment compared with healthy control subjects (Musen et al., 2012; Chen Y. C. et al., 2014; Hoogenboom et al., 2014). In this context, it is necessary to understand the potential mechanism of T2DM related cognitive impairment through brain network connectivity. The aim of our review is to summarize the relationship between T2DM related cognitive impairment and brain connectivity. We reviewed the possible pathophysiological mechanism behind this relationship through previous research evidence. We emphasize neuroimaging because this field provides a unique perspective to advance our understanding of cognitive impairment.

## T2DM Related Cognitive Impairment and Pathophysiology

**Figure 2:**
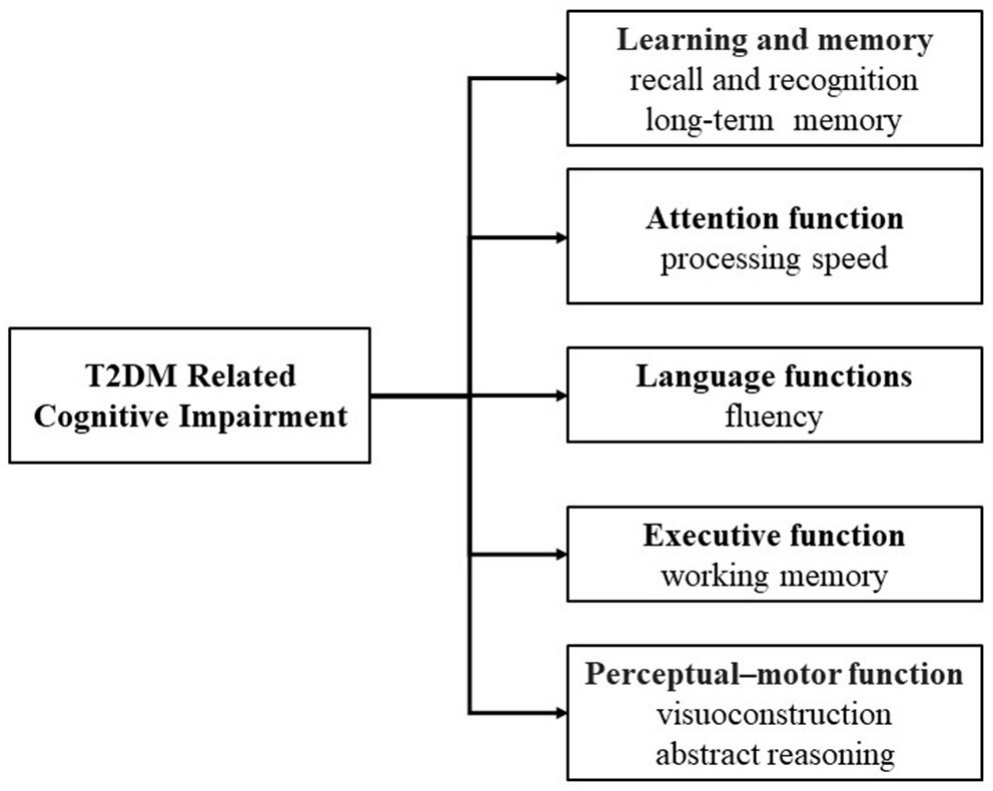
Domains of T2DM related cognitive impairment.

Compared with patients without diabetes, those with diabetes are more likely to experience cognitive decline, especially T2DM (Cheng et al., 2012; McCrimmon et al., 2012). T2DM is associated with cognitive impairment, mainly in verbal memory, processing speed and working memory (Messier, 2005; van den Berg et al., 2010). With increasing age, these may extend to other domains of cognition, for example, abstract reasoning and visuoconstruction (Awad et al., 2004; van den Berg et al., 2010) (Figure 2). Both glucose and insulin play an important role in the regulation of cognitive function, which is of great significance for the understanding of cognitive impairment in T2DM (Geijselaers et al., 2015).

The brain has a high demand for energy and less glycogen is stored in the brain, so glucose is the main source of energy for the brain (Jha and Morrison, 2018). Many brain regions, such as the hippocampus, are extremely sensitive to changes in blood glucose levels (Macauley et al., 2015; Ishibashi et al., 2016; Sarikaya et al., 2020). Insulin is also closely related to amyloid beta (Aβ), which may also lead to impaired brain glucose utilization (Bosco et al., 2011). However, it is not fully understood about the pathophysiological mechanism of the central nervous system changes caused by T2DM.

### T2DM and Neuroplasticity

Neuroplasticity refers to the ability of the nervous system to respond to internal or external stimuli by restructuring its structure and function (Cramer et al., 2011; Fu and Zuo, 2011). Neuroplasticity is a physiological phenomenon that occurs continuously at multiple levels, including intracellular processes (e.g., cell metabolism), cell-to-cell interactions (e.g., synaptic plasticity, axon growth and connectivity), neural circuits and brain structure (Cramer et al., 2011; Fu and Zuo, 2011). Long-term potentiation (LTP) and long-term depression (LTD) are important processes of signal transmission between neurons and are thought to represent the possible internal basis of sensory function, learning and memory (Malenka and Bear, 2004). There is separate evidence that insulin is a growth factor with neurotrophic properties that can affect neuroplasticity and consequently learning and memory (McIntyre et al., 2008; Banks et al., 2012). Diabetes could cause hippocampus atrophy, neuron loss and synaptic plasticity impairment. Several studies in animal models of diabetes focus on loss of neurons, and the cell proliferation decreased dramatically in the dentate gyrus (DG) of streptozotocin (STZ) - induced diabetic rats (Jackson-Guilford et al., 2000; Li et al., 2002; Stranahan et al., 2008; Ho et al., 2013). Several studies also provided evidence of the effects of diabetes on neuroplasticity in the hippocampus, which resulted in synapse loss and damage (Gispen and Biessels, 2000; Trudeau et al., 2004; Sims-Robinson et al., 2010).

DM is associated with impaired pathways related to neuroplasticity (Artola et al., 2005; Iwai et al., 2009; Reagan, 2012). Hyperglycemia and hyperinsulinemia affect the pathways of neuroplasticity (Trudeau et al., 2004; Bosco et al., 2011). Insulin degrading enzyme (IDE) can regulate the content of Aβ in neuronal cells. Hyperinsulinemia in DM can competitively inhibit IDE, thereby reducing the Aβ clearance (Yarchoan and Arnold, 2014). Hyperinsulinemia associated with T2DM can induce brain insulin resistance by causing a decrease in insulin receptor expression and receptor kinase activity, and ultimately promoting the development of Aβ and tau (Xie et al., 2002). It has also been demonstrated that Aβ indirectly promotes brain insulin resistance (Kim et al., 2011). Insulin resistance disrupts LTP which is the basic mechanism of memory integration (Kamal et al., 1999; Kim et al., 2011). In addition, higher plasma glucose levels can lead to the formation and accumulation of advanced glycation end products (AGEs) in diabetic patients and damage neuronal function through various mechanisms (Münch et al., 1998).

### T2DM and Energy Metabolism

The adult brain consumes a large amount of oxygen, accounting for about a quarter of the human body. Most energy is used for the propagation of action potentials and the maintenance of membrane potentials required for neuronal transmission (Attwell and Laughlin, 2001). The energy requirements of the brain vary greatly between different cells and between the resting state and the active state. In the resting state, the intrinsic activity of the brain accounts for most of the energy consumption (Raichle, 2010). The intrinsic activity may reflect the essence of brain function, such as maintaining the acquisition and maintenance of information for preconditioning, responding to and predicting environmental demands (Raichle, 2010). In contrast, the extra energy consumed by task evoked activity accounted for only a small fraction, suggesting the addition of task evoked activity on the basis of intrinsic activity (Huang, 2019). When performing tasks, the energy consumption is less, and the internal activities of the whole brain are inhibited to some extent, so the brain is more focused on task execution (Huang, 2019). In general, it has been observed that increased brain size and increased synaptic signaling between neurons leads to higher energy consumption (Sibson et al., 1998; Herculano-Houzel et al., 2006; Sherwood et al., 2006). Although this structure underlies more complex cognitive functions, it will impose a serious metabolic burden (Karbowski, 2007). The dynamic demand increases exponentially with the increase of capacity and connection distance (Chklovskii, 2004; Niven and Laughlin, 2008). Based on these limitations, the size of the nervous system can be reduced by reducing the number of neurons, by reducing the average size of neurons, or by laying out neurons so as to reduce the lengths of their connections, while maintaining adequate function (Laughlin and Sejnowski, 2003). From an evolutionary perspective, the organization of brain network may maximize information processing efficiency while minimizing cost (Laughlin and Sejnowski, 2003; Bullmore and Sporns, 2012). Therefore, a shortage of energy supply or excessive increase in demand may undermine the stability of the network organization and turn it into a dysfunctional configuration (Laughlin and Sejnowski, 2003; Bullmore and Sporns, 2012).

DM is considered the most common metabolic disorder worldwide, and this medical condition affects all organs and systems, including the brain (Gaspar et al., 2016). STZ - induced diabetic mice with cognitive decline had a peculiar metabolic phenotype in brain, mainly involving increased lactate level, decreased choline and energy metabolism as well as disrupted astrocyte-neuron metabolism (Zhang et al., 2020). Advanced stage of diabetes rats possessed peculiar metabolic phenotypes in serum and hippocampus, as characterized by decreases in tricarboxylic acid (TCA) cycle and amino acid and choline metabolism as well as disturbances in glutamate/GABA-glutamine cycle and astrocyte-neuron metabolism (Gao et al., 2019). Cerebral glucose levels are directly related to blood glucose levels; thus, changes in blood glucose impact the availability of glucose in the brain (Shestov et al., 2011). Affecting glucose levels leads to changes in brain function, which are largely related to neuroplasticity and memory/learning (Ma et al., 2015). Cellular glucose metabolism is mainly divided into glucose uptake and intracellular oxidative metabolism (Cisternas and Inestrosa, 2017). Insulin mediates the enhancement of glucose uptake and metabolism in neurons and glial cells (Cisternas and Inestrosa, 2017). In addition, in the brain, insulin is closely related to Aβ metabolism (McNay and Recknagel, 2011). Aβ in neurons has been specifically described to induce decreased glucose uptake and mitochondrial dyskinetic, both of which may lead to a reduction in neuronal energy production (Sadowski et al., 2004; Ahmed et al., 2015). Recent studies have shown that direct administration of Aβ to the brain can induce peripheral glucose intolerance, suggesting that Aβ can induce damage or directly impair neuronal metabolism in specific areas related to metabolic control (Clarke et al., 2015).

## Hypothesis: Brain Connectivity Link Neuroplasticity and Energy Metabolism

Structural connectivity refers to the study of the anatomical characteristics of the brain network according to subcortical white matter (WM), which is the basis for the interconnection between different cortical regions (Park et al., 2004). Diffusion MRI is to uses the water diffusion characteristics as a probe to estimate the brain fiber bundle (Basser et al., 1994). The analysis of themeso-microscopic level, decreased fractional anisotropy (FA) and elevated mean diffusivity (MD) generally indicate lower microstructural integrity within the neural structure (Winklewski et al., 2018). Other causes may also contribute to changes in diffusion measures, such as inflammation induced changes in fiber density or diameter, and the presence of complex crossing fibers and branches (Jeurissen et al., 2013; Winklewski et al., 2018). Although diffusion anisotropy theoretically reflects microscopic anatomy, spatial resolution obtained by MRI remains at the macroscopic level. On the macroscopic scale, structural connectivity measured by using *in vivo* neuroimaging reflects large-range fiber bundles inferred from diffusion MRI (Basser et al., 1994). The brain can be viewed as an anatomically separated network of local brain regions that communicate with other local regions through longer inter-regional white matter pathways (Hagmann et al., 2007; Tymofiyeva et al., 2014). The diffusion MRI changes observed in rats were correlated with immunohistochemical analysis, suggesting that diffusion MRI may be used to indirectly locate neuralplasticity (Blumenfeld-Katzir et al., 2011). Diffusion imaging in general to study neuroplasticity can provide deeper insight into the affected regions, connectivity between regions.

Functional connectivity (FC) attempts to establish the connection between different regions of the brain with the assistance of linear temporal correlation (Friston, 2011; Smith, 2012). Bullmore and springs concluded that brain functional connectivity can be analyzed by functional magnetic resonance imaging (fMRI), electroencephalogram (EEG), magnetoencephalogram (MEG) or multi electrode array (MEA) (Bullmore and Sporns, 2009). In particular, resting-state functional magnetic resonance imaging (rs-fMRI) has become a very important basis for FC analysis, after the discovery of spatially organized endogenous low-frequency fluctuations of blood oxygen level dependent (BOLD) signals (Biswal et al., 1995). Rs-fMRI research has been critical in revealing intrinsic, stable networks of the human brain, which are comprised of brain regions that appear consistently functionally connected even during rest (Fox et al., 2006). Functional connectivity infers that brain regions are more likely to be communicating with each other, if their highly correlated BOLD activity (i.e., have high FC) then they are frequently co-activated. However, the BOLD signal represents a non-specific proxy of activation, which is directly mediated by hemodynamic factors including changes in blood flow and oxygen content rather than by neural metabolism (Heeger and Ress, 2002). BOLD signals are likely to be dominated by changes in energy usage associated with synaptic currents and action potential propagation (Attwell and Laughlin, 2001). The brain uses most of its energy to support synaptic transmission, which is related to hemodynamic response (Logothetis et al., 2001; Niessing et al., 2005). Therefore, the high degree of connectivity of the ventral posterior region of the brain with other brain regions indicates an association suggests a close correlation between hemodynamic responses and neuronal synchronization (Fukunaga et al., 2008; Tomasi and Volkow, 2010). However, the energy budget of spontaneous fMRI signals is still largely unknown.

**Figure 3:**
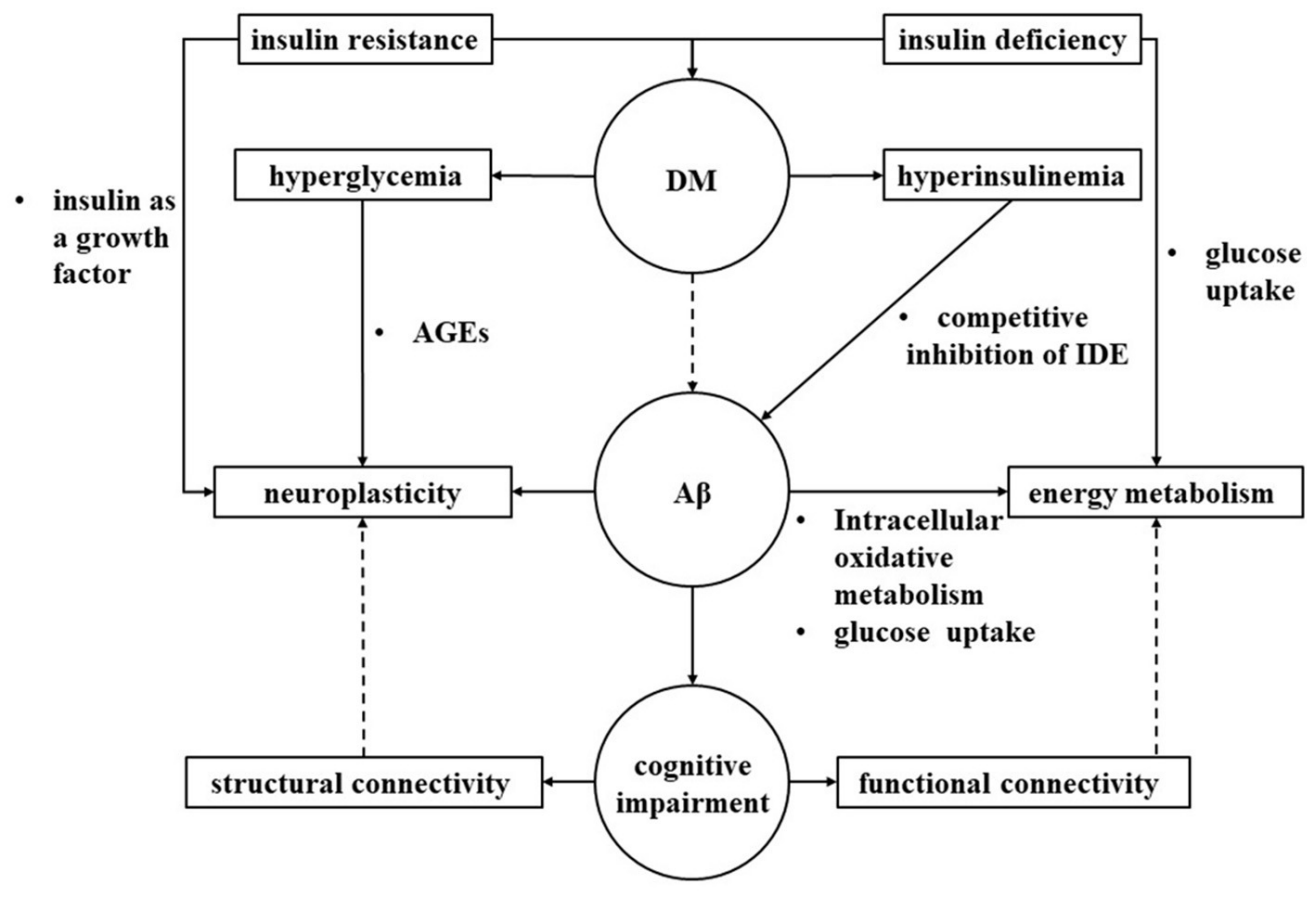
Interaction of brain connectivity, neural plasticity and energy metabolism. We hypothesized that diabetes may lead to abnormalities in neuroplasticity and energy metabolism in the brain through multiple pathological pathways, ultimately leading to cognitive impairment that may reflect to MRI structural connectivity and functional connectivity, respectively. IDE, insulin degrading enzyme; AGEs, advanced glycation end products; DM, Diabetes mellitus; Aβ, amyloid beta.

We hypothesized that cognitive impairment in diabetes may lead to abnormalities in neuroplasticity and energy metabolism in the brain, and those reflected to MRI structural connectivity and functional connectivity, respectively (Figure 3).

## T2DM Related Cognitive Impairment and Brain Connectivity

### T2DM Related Cognitive Impairment and Structural Connectivity

At the macroscopic scale, the brain can be viewed as a network composed of anatomically separated brain regions, and the white matter is an important basis for information transmission between brain regions (Park et al., 2004; Sporns et al., 2005). Diabetes may increases the chances of WM disease (Alexandrou et al., 2010; Katsumata et al., 2010; Ryu et al., 2014; Ogama et al., 2018). Neuroimaging studies have shown that changes in WM tract diffusion characteristics are associated with impaired glucose metabolism and cognitive dysfunction (Reijmer et al., 2013a; van Bussel et al., 2016a). However, the cognitive function of brain and white matter fiber tracking is not as simple as a one-to-one relationship. Due to the complexity of the brain network, it is difficult to attribute the microstructural damage of WM to specific cognitive functions. Preliminary reports on the significant correlation between WM fibers and cognitive function in patients with T2DM suggest that the detrimental effect of abnormal glucose metabolism on late brain function may be partly attributed to structural disruption of the WM tract, such as the bilateral frontal, bilateral cerebellum, temporary lobe, right caudate, bilateral cingulate gyrus, pons, and parahippocampal (Hsu et al., 2012). Several studies based on diffusion imaging for white matter tracking and network analysis have shown that T2DM is associated with reduced overall network properties and disrupted microstructural integrity (Hsu et al., 2012; Reijmer et al., 2013b; Zhang et al., 2014, 2019; Kim et al., 2016; van Bussel et al., 2016a; Xiong et al., 2016). In the above studies, the temporal lobe, prefrontal lobe and interconnected brain regions were significantly correlated with cognitive function in different regions.

The temporal lobe, especially the hippocampus, seems to be vulnerable to T2DM-induced structural damage. Gold et al. has shown that the medial temporal lobe may be the first region of the brain affected by T2DM and that individuals with poor metabolic control may be more significantly affected (Gold et al., 2007). T2DM is associated with damage to the microarchitecture and network in different regions of the temporal lobe and hippocampus, as well as corresponding damage to related fibers, which are closely related to memory formation and learning (van Bussel et al., 2016a; Xiong et al., 2016; Zhang et al., 2019). Interestingly, in human brain autopsy studies, increased Aβ and neurofibrillary tangles were found in the diabetic hippocampus, which is the hallmark pathology of Alzheimer's disease (Heitner and Dickson, 1997). It is speculated that the excessive development of glycation end products and insulin resistance may lead to the accumulation of Aβ and neurofibrils in DM, leading to macro-and micro-structural defects in these patients, leading to cognitive impairment (Münch et al., 1998; Kim et al., 2011).

In addition to the temporal lobe, the overall spread of gray matter (GM) and WM in the frontal lobe of DM showed abnormal microstructures. Van Bussel et al. found decrease in the white matter connection between the hippocampus and frontal lobe, which is associated with memory impairment in T2DM (van Bussel et al., 2016a). Furthermore, deficits in prefrontal WM integrity may be responsible for executive dysfunction and memory dysfunction in DM (Chua et al., 2008; Preston and Eichenbaum, 2013). Prefrontal WM damage is associated with the attention network and duration of disease in DM (Sanjari Moghaddam et al., 2019). It is possible that the attention deficit seen in diabetes over time is due in part to abnormalities in the prefrontal WM microstructure (Sanjari Moghaddam et al., 2019).

The thalamus appears to be an important candidate structure for cognitive decline associated with DM. Studies have shown thalamic deficits in vascular dementia and Alzheimer's disease can cause cognitive dysfunction in various areas, including attention, memory and motivation (Szirmai et al., 2002; Aggleton et al., 2016). The thalamus and its fiber-cortical tissue network are less, and the structural connectivity between thalamus and cortex is reduced, indicating that its microstructure is impaired (Chen et al., 2015). Thalamus is the main gateway that receives information and relays it to the frontal and temporal lobes (Chen et al., 2015; Yang et al., 2015). In the Papez circuit, it is connected to the middle temporal lobe and plays a major role in processing information related to memory and learning. Impaired integrity of the macromolecular protein pool in the frontal-striatal-thalamic circuit of DM is associated with HbA1c levels and vascular risk factors, as well as cognitive deficits in memory, executive function, information processing speed, and attention (Yang et al., 2015).

Graph theory is a powerful framework that allows the brain to be described as a complex network of interacting elements, the so-called connectome (Bullmore and Sporns, 2009). The application of graph theory provides a series of topological properties to evaluate integration, separation, thus providing unique insights into brain structure. The path length is a measure of network topology (Watts and Strogatz, 1998; Rubinov and Sporns, 2010; Bullmore and Sporns, 2012). Specifically, the path length between two nodes is the minimum number of edges that must pass from one node to another. The average shortest path length between all node pairs in the network is called the characteristic path length of the network. The path length estimates the potential of functional integration on the network, and shorter paths length mean stronger potential for network integration (Rubinov and Sporns, 2010). The average inverse shortest path length is a related measure known as global efficiency (Latora and Marchiori, 2001). Higher global efficiency reflects brain higher integration in processing information (Rubinov and Sporns, 2010). The clustering coefficient reflects network segregation, which increases with the increase of the number of connections between neighboring nodes or reduction in total number of nodes within a network (Rubinov and Sporns, 2010). Healthy brain is organized according to highly isolation and integration, which is also the basis of normal cognitive function (Sporns et al., 2004). Tijms et al. investigated the link between amyloid pathology and network measures in cognitively healthy individuals, revealing that lower cerebrospinal fluid (CSF) Aβ42 levels were associated with lower connectivity density, reduced clustering, and higher path length (Tijms et al., 2013). Studies with structural brain connectomes have shown that the amyloid effects on the white matter to networks are associated with early neurodegeneration (Pereira et al., 2018). The pattern of disease transmission is potentially limited by connectome, which can provide an important perspective on how network topology shapes the neural response to early injury (Seeley et al., 2009; Raj et al., 2012).

Previous studies based on graph theory have reported the disruption of brain network integration in T2DM, which is reflected in the longer characteristic path length or lower global efficiency (Reijmer et al., 2013b; Kim et al., 2016; Zhang et al., 2016, 2019). The longer path length indicates that the information transmission and integration between long-distance neurons are impaired, resulting in reduced communication efficiency (Sporns and Zwi, 2004). In other words, remote brain regions seem unable to interact effectively due to the effects of T2DM. However, the clustering coefficient which represents network segregation did not show a consistent pattern in T2DM (Reijmer et al., 2013b; Kim et al., 2016; Zhang et al., 2019). Specifically, Reijmer et al. found that the clustering coefficient was lower in well-controlled T2DM compared to healthy controls, while Kim et al. suggested that patients with poorly controlled diabetes did not differ in clustering coefficients compared with controls (Reijmer et al., 2013b; Kim et al., 2016). Moreover, Zhang et al. reported that the clustering coefficient of patients without clinical vascular complications was higher (Zhang et al., 2019). There is also a difference in local efficiency, which is a measure of the fault tolerance of the network (Zhang et al., 2016, 2019). Zhang et al. found that the local efficiency of T2DM was lower than healthy controls, but Zhang et al. suggested that there was no difference between them (Zhang et al., 2016, 2019). Obviously, more studies are needed to draw reliable conclusions about the effects of T2DM on the structural topological properties of the local networks.

### T2DM Related Cognitive Impairment and Functional Connectivity

A range of disorders associated with cognitive impairment have previously been observed to be associated with impaired connectivity of the default mode network (DMN), including schizophrenia, depression, and neurodegenerative disorders (Broyd et al., 2009). In resting-state functional imaging of Alzheimer's disease, the changes of DMN mainly focus on the decrease of connectivity and task-induced inactivation, which is more obvious than normal aging (especially in the hippocampus which is an important part of the decreased connectivity with other DMN regions) (Buckner et al., 2008; Damoiseaux et al., 2008; Mevel et al., 2011; Sala-Llonch et al., 2015). Since DMN is an important brain network involved in cognitive processes, the relationship between DMN and cognitive impairment in DM has also received extensive attention.

The changes of DMN has been observed in DM with cognitive impairment, but the reason is uncertain. Cognitive impairment is characterized by reduced DMN connectivity in the hippocampus and posterior cingulate gyrus (Broyd et al., 2009). Studies have found evidence of neuronal dysfunction associated with preclinical memory during episodic memory encoding in T2DM patients (Tromp et al., 2015; Wood et al., 2016). Similarly, episodic memory impairment in T2DM is associated with reduced DMN inactivation and reduced connectivity between the anterior and posterior DMN (Marder et al., 2014; Chen et al., 2016). Impaired cognitive ability in DM is associated with local changes in spontaneous brain activity and decreased connectivity between DMN (Cui et al., 2014; Peng et al., 2016). In aging, the connectivity of DMN has been associated with the structural integrity of gray matter and white matter in aging-related nodal regions (Vidal-Piñeiro et al., 2014). The hippocampus is susceptible to T2DM and aging, and the prefrontal lobe is a vulnerable brain region in diabetic patients, and the reduced number of connections between the hippocampus and the frontal lobe may be associated with diabetic cognitive impairment (Du et al., 2006). Functional connectivity in DMN critical regions of T2DM, including the posterior cingulate gyrus and medial frontal gyrus, is associated with lower white matter integrity in the cingulate tract, and also indicates interactions among prefrontal, parietal, and temporal regions (Jones et al., 2013; Hoogenboom et al., 2014). Moreover, T2DM patients, especially T2DM patients with cognitive impairment, have impaired connectivity within and between networks, mainly involving the bilateral posterior cerebellum, the right insula, the DMN and the control network (CON) (Yang et al., 2016). In the elderly with cognitive health, increased brain amyloid load and lower CSF Aβ42 levels are associated with decreases in functional connectivity in regions that belong to the DMN (Sheline et al., 2010; Wang et al., 2013). The loss of regions pertinent to the DMN is in line with the hypothesis that neurodegenerative disorders tend to target the more well-connected brain regions (Seeley et al., 2009). These findings suggest that common mechanisms may underlie the structural and DMN connectivity abnormalities observed in DM, but more research is needed to further investigate the underlying causes of DMN dysfunction using combined structural and functional indicators.

Changes in brain function in T2DM are associated with neuropsychological task performance (Xia et al., 2013; Chen Y. et al., 2014; Zhang et al., 2015; Liu et al., 2016; van Bussel et al., 2016b; Yang et al., 2016; Wang et al., 2017). Task-state functional magnetic resonance studies have shown that a lower task burden is accompanied by a decrease in brain activation in T2DM, but as task burden increases, brain activation increases to normal levels (He et al., 2015; Huang et al., 2016). Increased activation accompanied by improved task performance may be related to neurological compensatory mechanisms (Morcom and Johnson, 2015). Evidence for an inverse increase in functional connectivity was also found in a magnetoencephalographic study of diabetic patients without retinopathy (Qi et al., 2010). Prior to the onset of cognitive decline in clinical manifestations in diabetic patients, which may be at an early stage of the lesion or caused by local brain compensation, functional reorganization of the brain network may have begun to work to offset the slight reduction in cognitive performance (van Bussel et al., 2016b). We speculate that when functional reorganization fails, it will lead to functional network disorder and cognitive impairment.

Although widely distributed functional dysconnectivity in many brain regions have been demonstrated in previous rs-fMRI studies, the dysconnectivity within these regions has been largely considered to represent independent abnormalities in isolation from each other (Xia et al., 2013; Cui et al., 2014; Wang et al., 2014, 2017; Peng et al., 2016). fMRI network analysis based on graph theory can reveals the intrinsic network dysfunction of T2DM related cognitive impairment (van Bussel et al., 2016b; Chen et al., 2017; Liu et al., 2018; Qin et al., 2019; Xu et al., 2019). Although the results on cognitive function are varied, Dai and He review that increased characteristic path length and decreased number of hubs are the most consistent findings in patients with cognitive impairment (Dai and He, 2014). However, there is also some empirical evidence that higher clustering and over integration can lead to cognitive deterioration (Alexander-Bloch et al., 2013; Giessing et al., 2013). These pathological areas may increase the number of connections with nearby neighbors and form a disorder along the path of network transmission of information (Pereira et al., 2018). Possibly, deviations from the small world architecture in either direction can potentially degrade performance.

Graph theory analysis shows that the functional brain network of type 2 diabetes still has the characteristics of small world (Xu et al., 2019). However, the whole brain networks show that the normalized clustering coefficient and characteristic path length have changed, indicating that the functional brain network of DM has changed (Qin et al., 2019; Xu et al., 2019). The changes of nodal topological alterations were mainly concentrated in the frontal lobe, temporal lobe and cingulate gyrus, which are related to cognitive ability and HbA1c level (Qin et al., 2019). Those brain regions are consistent with DMN regions, indicating that they may be caused by DMN damage (Brier et al., 2014). Some studies have found that the network topology of DM is abnormal, but cognition still normal (van Bussel et al., 2016b; Qin et al., 2019; Xu et al., 2019). Even DM show higher standardized clustering coefficient and higher local efficiency (van Bussel et al., 2016b; Qin et al., 2019; Xu et al., 2019). It has been suggested that compensation mechanisms in whole brain networks appear at an early stage (van Bussel et al., 2016b). Some scholars have found that aging with normal cognitive function have more functional connections, which may be attributed to the hypothesis of “brain reserve” and “acceleration” (Cohen et al., 2009; Hedden et al., 2009; Lim et al., 2014). Specifically, the brain reserve hypothesis is that individual differences in occupation, education, physical, mental, and social activities provide reserve against brain pathology (Stern, 2002; Scarmeas and Stern, 2004). Bigger brains may tolerate more loss before exhibiting impaired function because of higher number of healthy synapses or neurons (Tucker and Stern, 2011). Additionally, brain reserve focuses more on the “software” in which brain reserve allows individuals to have higher neural efficiency, greater neural ability, and the ability to compensate by recruiting additional brain regions (Tucker and Stern, 2011). The acceleration hypothesis that has been cited in the literature to explain increased brain activation and glucose metabolism with increasing AD risk, but not atrophy or cognitive loss (Dickerson et al., 2004; Jacobs et al., 2012; Johnson et al., 2014). However, higher basal metabolism may exacerbate Aβ deposition, eventually leading to cognitive decline (Cohen et al., 2009; Johnson et al., 2014). In addition, the duration of diabetes may be related to the observed changes in brain function, so the duration of the disease is linearly related to the decline in cognitive ability, which needs to be further confirmed by longitudinal functional magnetic resonance imaging (Tuligenga et al., 2014; Wood et al., 2016).

## Prospect

MRI structural and functional connectivity analyses can help identify drivers of cognitive dysfunction. Altered brain connectivity maybe used as a biomarker to distinguish between cognitive functions caused by diabetes or other factors. Changes in hemodynamic reactivity and neurovascular coupling in patients with DM lead to reduced vascular reactivity, which may lead to impaired BOLD signals (Venkat et al., 2016; Yan et al., 2020). Drugs associated with cardiovascular disease significantly affect cerebral perfusion, and they may also play a role in neuroplasticity (Krimer et al., 1998; Honey and Bullmore, 2004; Chollet, 2013). Therefore, the impact of drugs is an issue that needs to be explored in future studies. The role of gender in functional brain changes remains to be explored (Ingalhalikar et al., 2014). Sex hormones affect the homeostasis of glucose, so the question of whether gender differentially affects cognition and brain activation in patients with T2DM deserves further exploration (Varlamov et al., 2014).

## Conclusion

In light of the current research evidence, structural and functional brain connectivity changes are associated with a wide range of cognitive functional alterations in patients with T2DM. Although the causes of cognitive impairment in T2DM remain incompletely understood, disrupted neuroplasticity and impaired energy metabolism in the brain are considered possible candidates. MRI structural and functional connectivity analyses reflect associations between brain regions and may be related to information transmission and synchronization. Cognitive impairment in patients with T2DM may lead to abnormal neuroplasticity and energy metabolism in the brain, which are reflected in MRI structural connectivity and functional connectivity. The temporal lobe, prefrontal lobe, thalamus, and DMN appear to be important regions influencing cognitive function. Since the pattern of disease transmission may be limited by the connectome, this could provide an important perspective to explore how T2DM affects cognition at the brain network level. In addition, most of the current studies on T2DM do not consider the effects of drugs, sex hormones, and microvascular damage on cerebral hemodynamic reactivity and neurovascular coupling, which are issues to be explored in future imaging studies. In conclusion, we believed that T2DM related cognitive impairment may be an overall impairment of brain networks.

## Author Contributions

HL, RH, and GHL made equal contributions to this review, analyzed references, and drafted manuscript. HZ and JCL contributed to the topic conception, manuscript revision, and decision to submit for publication. The remaining authors contributed to reference collection and proofreading the language. All authors read the final manuscript and approved it for publication.

## Funding

This work was supported by Hunan Provincial Science and Technology Innovation Program of China [Grant No. 2017SK50203 (HZ)], Scientific Research Fund Project of Hunan Provincial Health Commission [Grant No. 20201911 (HZ)], Science and Technology Project of Hengyang [Grant No. HCJZ20206720 (JCL)], Hunan Provincial Natural Science Foundation of China [Grant No. 2018JJ2357 (GHL)], and Hunan Provincial Innovation Foundation for Postgraduate [Grant No. CX203YSC066 (TQY)].

## Conflict of Interest

The authors declare that the research was conducted in the absence of any commercial or financial relationships that could be construed as a potential conflict of interest.

## Publisher's Note

All claims expressed in this article are solely those of the authors and do not necessarily represent those of their affiliated organizations, or those of the publisher, the editors and the reviewers. Any product that may be evaluated in this article, or claim that may be made by its manufacturer, is not guaranteed or endorsed by the publisher.
