# Supplementary material for: Altered Structural and Functional MRI Connectivity in Type 2 Diabetes Mellitus Related Cognitive Impairment: A Review
Source: Front Hum Neurosci. 2022 Jan 6;15:755017. doi: 10.3389/fnhum.2021.755017 (PMC8770326; doi:10.3389/fnhum.2021.755017)
Supplement: Supplementary file 1 [file Table_1.DOCX]

**Table S1**

Cognitive relevant brain connectivity changes in patients with type 2 diabetes mellitus

|  | References | Number  (control/patient) | Age range  (year) | Method | Results and interpretation |
| --- | --- | --- | --- | --- | --- |
| Structural connectivity | Reijmer, Y.D., et al. (2013) | 50/55 | 70.9 ± 4.5  70.9 ± 4.4 | graph theory | The mean clustering coefficient and the global efficiency of the network were decreased; the shortest path length was increased. These changes were related to slowing of information processing speed. |
|  | Zhang, Y., et al. (2019) | 57/57 | 54.46 ± 6.93  55.98 ± 8.19 | graph theory | The characteristic path length was negatively correlated with the cognitive state; the clustering coefficients, the nodal global efficiency in the right hippocampus and the superior pole of the right temporal were positively correlated with the cognitive state. |
|  | Kim, D.J., et al. (2016) | 20/20 | 54.3 ± 2.4  54.6 ± 2.3 | graph theory | Poorer glycemic control is associated with lower efficiency and longer connection paths and leads to mental decline and cognitive impairment. |
|  | Zhang, J., et al. (2016) | 886/163 | 66.5 ± 6.9  65.5 ± 6.7 | graph theory | Global network properties and the nodal efficiency of the right rolandic operculum both had positive correlations with executive function. |
|  | van Bussel, F.C. (2016) | 38/40 | 61.1 ± 9.5  62.7 ± 6.7 | seed-based | The white matter connections between the hippocampus and frontal lobe was related to memory decrements. |
| Functional connectivity | Chen, Y.C., et al. (2015) | 39/38 | 57.1 ± 6.4  58.6 ± 7.3 | seed-based | Thalamus connectivity with the right middle temporal gyrus correlated with impaired cognitive performances. |
|  | Liu, D., et al. (2016) | 25/25 | 52.08 ± 3.46  52.24 ± 4.78 | seed-based | The functional connectivity between the right fusiform gyrus and the middle temporal gyrus was negatively correlated with working memory. |
|  | Liu, D., et al. (2018) | 47/47 | 57.36 ± 5.42  58.66 ± 6.87 | seed-based | The left anterior cingulate gyrus, right precentral gyrus, and bilateral lateral occipital cortices were associated with the disruption of visual information acquisition and goal-directed action execution. |
|  | van Bussel, F.C., et al. (2016) | 45/47/47 | 60.7 ± 6.5  61.0 ± 6.7  61.0 ± 6.7 | graph theory | Compared with control participants and pre-diabetic patients, the higher normalized cluster coefficient and higher local efficiency of T2DM may be attributed to an earlier stage of structural brain damage, where compensatory mechanisms, such as functional reorganization of networks. |
|  | Yang, S.Q., et al. (2016) | 19/19/19 | 60.21 ± 5.35  59.53 ± 6.17  61.95 ± 5.93 | graph theory | The bilateral posterior cerebellum, the right insula, the posterior cingulate cortex were associated with cognitive impairment. |
|  | Zhang, H., et al. (2015) | 14/14 | 60.1 ± 9.9  61.7 ± 8.1 | seed-based | Intranasal insulin administration to regulating memory and complex cognitive positively correlated with the resting-state connectivity between the hippocampal regions and the medial frontal cortex, right inferior parietal cortex, right anterior and posterior cingulate cortex. |
|  | Chen GQ., et al. (2017) | 24/12 | 66.4 ± 5.5  67.3 ± 4.7 | graph theory | Characteristic path length showed a negative correlation with MMSE scores and global efficiency showed a positive correlation. |
|  | Qin C, et al. (2019) | 30/30 | 48.87 ± 0.98  51.77 ± 1.42 | graph theory | The right middle occipital gyrus, right posterior cingulate gyrus was negatively correlated with cognitive impairment; the right gyrus rectus, left amygdala, right cerebellum 3 was positively correlated with cognitive impairment. |

T2DM, type 2 diabetes mellitus; DMN, default mode network. ICA, independent components analysis; MMSE, mini mental state examination
